# Short‐term cost‐utility of degludec versus glargine U100 for patients with type 2 diabetes at high risk of hypoglycaemia and cardiovascular events: A Canadian setting (DEVOTE 9)

**DOI:** 10.1111/dom.13730

**Published:** 2019-04-14

**Authors:** Richard F. Pollock, Simon Heller, Thomas R. Pieber, Vincent Woo, Jens Gundgaard, Nino Hallén, Maria Luckevich, Deniz Tutkunkardas, Bernard Zinman, On Behalf of the DEVOTE Study Group

**Affiliations:** ^1^ Ossian Health Economics and Communications GmbH Basel Switzerland; ^2^ University of Sheffield Sheffield UK; ^3^ Medical University of Graz Graz Austria; ^4^ University of Manitoba Winnipeg Manitoba Canada; ^5^ Novo Nordisk A/S Søborg Denmark; ^6^ Novo Nordisk Canada Inc Mississauga Ontario Canada; ^7^ Lunenfeld‐Tanenbaum Research Institute, Mount Sinai Hospital, University of Toronto Toronto Ontario Canada

**Keywords:** cost‐effectiveness, insulin analogues, insulin therapy, pharmaco‐economics

## Abstract

**Aims:**

To evaluate the short‐term cost‐effectiveness of insulin degludec (degludec) vs insulin glargine 100 units/mL (glargine U100) from a Canadian public healthcare payer perspective in patients with type 2 diabetes (T2D) who are at high risk of cardiovascular events and hypoglycaemia.

**Materials and methods:**

A decision analytic model was developed to estimate costs (2017 Canadian dollars [CAD]) and clinical outcomes (quality‐adjusted life years [QALYs]) with degludec vs glargine U100 over a 2‐year time horizon. The model captured first major adverse cardiovascular event, death, severe hypoglycaemia and insulin dosing. Clinical outcomes were informed by a post hoc subgroup analysis of the DEVOTE trial (NCT01959529), which compared the cardiovascular safety of degludec and glargine U100 in patients with T2D who are at high cardiovascular risk. High hypoglycaemia risk was defined as the top quartile of patients (n = 1887) based on an index of baseline hypoglycaemia risk factors.

**Results:**

In patients at high hypoglycaemia risk, degludec was associated with mean cost savings (CAD 129 per patient) relative to glargine U100, driven by a lower incidence of non‐fatal myocardial infarction, non‐fatal stroke and severe hypoglycaemia, which offset the slightly higher cost of treatment with degludec. A reduced risk of cardiovascular death and severe hypoglycaemia resulted in improved effectiveness (+0.0132 QALYs) with degludec relative to glargine U100. In sensitivity analyses, changes to the vast majority of model parameters did not materially affect model outcomes.

**Conclusion:**

Over a 2‐year period, degludec improved clinical outcomes at a lower cost as compared to glargine U100 in patients with T2D at high risk of cardiovascular events and hypoglycaemia.

## 1 INTRODUCTION

Diabetes is a major global public health concern because of its high prevalence and association with morbidity, mortality and economic burden.^1^ In the past decade, the age‐standardized prevalence of diabetes in Canada has increased by 37% and is expected to continue to rise, reaching 5 million Canadians (12.1%) by 2025, driven by the increase in and the aging of the Canadian population.^2^, ^3^ As a result, approximately 2.6 million Canadians are currently living with diagnosed diabetes.^1^ In the 5 years between 2004 and 2008, diabetes was reported as an underlying or contributing cause of death in more than 120 000 Canadians, corresponding to 10.6% of all deaths reported during that period.^4^ Cardiovascular disease has been identified as the most common co‐existing condition reported on death certificates, appearing nine out of ten times when diabetes was listed as the underlying cause, and underpinning four out of ten deaths where diabetes contributed.^4^ Canada is currently one of the top ten countries in terms of expenditure on diabetes, when considering both total expenditure (15 billion international dollars [ID], used by economists to compare values of different currencies) and mean expenditure per person (over 5700 ID).^1^

Hypoglycaemia has considerable negative effects on patient quality‐of‐life, while posing a significant economic burden through increased healthcare resource utilization and loss of productivity.^5^, ^6^ The unpleasant symptoms of hypoglycaemia can result in significant anxiety about the possibility of future events, and fear of hypoglycaemia among both patients and clinicians can adversely affect diabetes management and clinical outcomes.^7^, ^8^ Severe hypoglycaemic events are associated with an increased risk of adverse clinical outcomes, including major adverse cardiovascular events (MACE), major microvascular events, dementia (in elderly patients) and death.^9^, ^10^ Several factors increase the risk of severe hypoglycaemia, including age, diabetes treatment regimen, duration of diabetes, presence of comorbidities, history of previous severe hypoglycaemic episodes and impaired awareness of hypoglycaemia.^11^, ^12^

Insulin degludec (degludec) is a basal insulin with an ultra‐long duration of action and a stable glucose‐lowering profile.^13^ Randomized controlled trials have confirmed that similar improvements in glycaemic control can be achieved, with fewer hypoglycaemic episodes, across a broad spectrum of patients with diabetes receiving degludec vs insulin glargine 100 units/mL (glargine U100).^14^, ^15^ Thus, it is of interest to investigate cost‐effectiveness in patients at high risk of hypoglycaemia who might be expected to benefit most from treatment with degludec compared with glargine U100. In the DEVOTE trial, a cardiovascular outcomes trial (CVOT) that compared the cardiovascular safety of degludec with that of glargine U100, degludec was non‐inferior to glargine U100 with respect to the incidence of MACE, but was associated with significantly fewer episodes of severe hypoglycaemia at similar levels of glycaemic control.^16^

A cost‐utility analysis (CUA) is a cost‐effectiveness analysis (CEA) that compares the costs of new interventions with their outcomes measured in utility units, most commonly, the quality‐adjusted life year (QALY), and captures both quantity‐ and quality‐of‐life over a specified time horizon. Health economic analyses, including CUAs, are an important tool to assist healthcare professionals in deciding how best to allocate resources efficiently between therapy areas and interventions to achieve maximum healthcare gains within a limited budget.^17^

Prior to the DEVOTE trial, CEAs of degludec compared with glargine U100 captured the effects of hypoglycaemia rates and insulin dosing over a short‐term (1‐year) time horizon in patients with type 1 (T1D) or type 2 (T2D) diabetes, based on the phase 3 clinical trial programme.^18^, ^19^, ^20^, ^21^, ^22^, ^23^ The DEVOTE trial provided an opportunity to evaluate randomized, double‐blind clinical trial data, including cardiovascular endpoints and death, in addition to severe hypoglycaemia rates and insulin dosing, to provide health economic analyses of degludec vs glargine U100 over a 2‐year time horizon without extrapolation.

The aim of the present post hoc analysis was to evaluate, from a Canadian public healthcare payer perspective, the short‐term cost‐utility of degludec vs glargine U100 in patients with T2D who are at high risk of hypoglycaemia and cardiovascular events.

## 2 MATERIALS AND METHODS

### 2.1 DEVOTE trial design

The trial design and primary results of the DEVOTE trial have been published previously.^16^, ^24^ In brief, the DEVOTE trial was a randomized, double‐blind CVOT that compared the cardiovascular safety of degludec and glargine U100 in patients with T2D at high cardiovascular risk (refer to Supporting Information, Methods for additional details). Patients (*N* = 7637) were randomly assigned 1:1 to receive once‐daily degludec (100 units/mL) or glargine U100, in addition to standard care. The primary endpoint in the DEVOTE trial was time to first MACE, a composite of cardiovascular death, non‐fatal myocardial infarction (MI) or non‐fatal stroke. Severe hypoglycaemia was self‐recorded in the DEVOTE trial and was defined according to the American Diabetes Association definition as an event requiring third‐party assistance.^25^ Non‐severe hypoglycaemia was not recorded. Events of MI, stroke, death and severe hypoglycaemia were independently adjudicated by the DEVOTE Event Adjudication Committee.

The DEVOTE trial was conducted in accordance with the provisions of the Declaration of Helsinki and the International Conference on Harmonisation Good Clinical Practice Guidelines, the DEVOTE protocol was approved by the independent ethics committee or institutional review board at each trial centre and written informed consent was obtained from each patient before any trial‐related activities.

### 2.2 Cost‐utility model overview

A decision analytic model built in Microsoft Excel 2016 was used to evaluate the short‐term cost‐utility of degludec vs glargine U100, informed by data concerning clinical outcomes and baseline characteristics for the DEVOTE subgroup at high risk of hypoglycaemia (subgroup defined using an index of established hypoglycaemia risk factors; refer to Section 2.3). The analysis was conducted over a 2‐year time horizon from a Canadian public healthcare payer perspective. Details of the model structure have been published previously, alongside the findings of a short‐term CUA informed by data from the DEVOTE basal‐bolus subgroup.^26^ In brief, a short‐term cohort model with two annual time cycles captured first MACE, death from other causes (ie, any cause except that of first MACE), severe hypoglycaemia and insulin dosing based on rates from the trial (Table 1 and Figure S1). Treatment differences were estimated using hazard, rate and dose ratios from regression analyses. Treatment effects and dose differences were captured only in the case of a statistically significant difference between treatment arms; otherwise, event rates and doses from the glargine U100 arm were modelled in both treatment arms. An annual discount rate of 1.5% was applied to costs and clinical outcomes in the second annual time period, in line with recommendations from the Canadian Agency for Drugs and Technologies in Health.^27^

**Table 1:** Input parameters: Clinical outcomes from the DEVOTE subgroup at high risk of hypoglycaemia

	Degludec/glargine U100 ratio	SE	95% CI	*P*‐value	Degludec^a^	Glargine U100
Complications					*Event rate (events/PYO)*	
First MACE	0.757	0.13	0.577; 0.992	0.044^b^	0.0493^c^	0.0651^d^
Severe hypoglycaemia	0.557	0.182	0.390; 0.796	0.0013^b^	0.0608	0.1092
Death from other causes	0.721	0.235	0.455; 1.142	0.1634^b^	0.0232	0.0232
Insulin dose					*Mean dose (units)*	
Basal insulin						
Baseline	N/A				46.90	46.90^d^
12 Months	1.049	0.022	1.005; 1.096	0.0306	61.05	58.20^d^
24 Months	1.087	0.030	1.024; 1.154	0.0060	67.72	62.30^d^
Bolus insulin						
Baseline	N/A				30.21	30.21^e^
12 Months	0.959	0.033	0.899; 1.024	0.2137	48.16	48.16^e^
24 Months	0.946	0.047	0.863; 1.037	0.2379	59.04	59.04^e^

Abbreviations: CI, confidence interval; glargine U100, insulin glargine 100 units/mL; MACE, major adverse cardiovascular event; MI, myocardial infarction; N/A, not applicable; *P*, *P*‐value; PYO, patient‐year of observation; SE, standard error.

a Model inputs for the degludec arm were estimated by applying the degludec/glargine U100 ratio to the corresponding glargine arm value, if there was a significant difference between treatments (*P* ≤ 0.05). Otherwise, the glargine U100 value (event rate or mean dose) was assumed in the model for both treatment arms.

b *P*‐value refers to a two‐sided test of degludec/glargine U100 ratio = 1.0.

c Non‐fatal MI: 41.5%; non‐fatal stroke: 24.4%; cardiovascular death: 34.1%.

d Non‐fatal MI: 42.4%; non‐fatal stroke: 26.3%; cardiovascular death: 31.3%. Modelled as the arithmetic mean of the start‐ and end‐of‐year glargine U100 basal doses, adjusted for survival in each annual time period.

e As described in footnote^d^, this was repeated for bolus insulin, but mean annual bolus dose was estimated by multiplying the proportion of patients receiving bolus insulin at baseline, 12 months or 24 months by the mean bolus dose for each time point.

### 2.3 Simulated cohort and treatment effects

In this analysis, the subgroup at high risk of hypoglycaemia was defined as the top quartile of patients in the DEVOTE trial, based on an index of established risk factors for severe hypoglycaemia at baseline, including age, diabetes duration, HbA1c, sex and insulin regimen. This internal index of high risk of severe hypoglycaemia was estimated using a Cox regression model of time to first event of severe hypoglycaemia for the full DEVOTE trial population (Table S1). At baseline, the DEVOTE subgroup at high risk of hypoglycaemia (n = 1887; degludec, n = 956; glargine U100, n = 931) was 64.4% female, had a mean (standard deviation [SD]) age of 68.1 (7.3) years, a mean (SD) HbA1c of 8.64% (1.73%) [71 mmol/mol (19 mmol/mol)] and a mean (SD) diabetes duration of 23.5 (9.3) years (Table S2). The majority of patients (88.3%) in the subgroup were using a basal‐bolus insulin regimen at baseline. Mean (SD) observation time for this subgroup was 1.96 (0.41) years.

Analyses were conducted to estimate treatment effects (degludec vs glargine U100) in the DEVOTE subgroup at high risk of hypoglycaemia for the following endpoints: time to first MACE, death from other causes, number of severe hypoglycaemic events, insulin dose and change in HbA1c from baseline to the 24‐month visit (refer to Supporting Information, Methods for additional details). The resulting relative rates for MACE, death from other causes and severe hypoglycaemia (hazard or rate ratios) were applied to the event rates (events/patient‐year of observation) observed in the DEVOTE trial in the glargine U100 arm, to derive event rates for input into the model for the degludec arm, if there was a statistically significant difference between treatment arms (Table 1).

Event rates for MACE were distributed among the individual MACE components (cardiovascular death, non‐fatal MI and non‐fatal stroke), based on the observed distribution of MACE components in the corresponding treatment arm in the DEVOTE trial. Adopting a conservative approach, subsequent cardiovascular events after first MACE were not incorporated into the model. Annual survival was estimated using data for cardiovascular death from first MACE and death from other causes, applied in each of the two annual time periods. Events rates for non‐fatal MI, non‐fatal stroke and severe hypoglycaemia were applied to the surviving cohort in the two annual time periods. In the glargine U100 arm, the basal insulin dose was modelled as the arithmetic mean of the start‐ and end‐of‐year glargine U100 basal doses to approximate the area under the curve, adjusted for survival in each annual time period. This was repeated for bolus insulin, but the mean annual bolus dose was estimated by multiplying the proportion of patients receiving bolus insulin at baseline, 12 or 24 months by the mean bolus dose for each time point. In the case of significant differences between treatment arms, the dose ratio (degludec/glargine U100) was applied to the mean annual glargine U100 basal or bolus dose to derive corresponding doses in the degludec arm (Table 1).

HbA1c was not captured in the model as there was no significant difference in change in HbA1c from baseline to 24 months with degludec vs glargine U100 for the DEVOTE subgroup at high risk of hypoglycaemia (degludec, −0.96%; glargine U100, −0.99%; estimated treatment difference, 0.03% (95% CI −0.08; 0.15; *P* = 0.603), as would be expected in a treat‐to‐target trial.

### 2.4 Costs, utilities and time horizon

Treatment unit costs were based on Canadian list prices (Table 2).^28^ Glargine U100 has been available in Canada since November 2004, well‐established public‐wide reimbursement. Degludec was more recently approved in Canada (August 2017) and public coverage grew throughout 2018, reaching similarly broad levels of reimbursement (correct as of February 2019). Based on publicly available list prices for Ontario, the basal insulin market share for glargine U100, the market leader, was roughly 10‐fold larger than that of degludec in 2018, although the market landscape was changing during that period.

**Table 2:** Input parameters: Costs and utilities

Parameter	Value	Unit	Source
Treatment costs (unit price)			
Glargine U100^a^	0.0727	CAD	ODBF 2017^28^
Degludec^b^	0.0835	CAD	NNCI Ontario wholesale price
IAsp^c^	0.0507	CAD	ODBF 2017^28^
Needle^d^	0.4855	CAD	ODBF 2017^28^
SMBG test strip^e^	0.8173	CAD	ODBF 2017^28^
Complication costs			
Non‐fatal MI, Year 1	19 806.63	CAD	CADTH 2017^29^
Non‐fatal MI, Year 2	3097.33	CAD	CADTH 2017^29^
Non‐fatal stroke, Year 1	26 978.80	CAD	CADTH 2017^29^
Non‐fatal stroke, Year 2	3743.24	CAD	CADTH 2017^29^
Cardiovascular death	0	CAD	CADTH 2017^29^
Severe hypoglycaemia	2178.62	CAD	CADTH 2017^29^
Utilities			
Base	0.785	Utility	Clarke et al. 2002^30^
MI	−0.055	Disutility	Clarke et al. 2002^30^
Stroke	−0.164	Disutility	Clarke et al. 2002^30^
Severe hypoglycaemia	−0.0592	Disutility	Harris et al. 2014^6^

*Note*: Complication costs were inflation‐adjusted to 2017 CAD using the Canadian Health and Personal Care component of the Consumer Price Index. Treatment costs include a CAD 8.83 dispensing fee per pack alongside an 8% pharmacy mark‐up with the exception of needles (10% mark‐up) and SMBG test strips (no mark‐up).

Abbreviations: CAD, Canadian dollar; CADTH, Canadian Agency for Drugs and Technologies in Health; IAsp, insulin aspart; glargine U100, insulin glargine 100 units/mL; MI, myocardial infarction; NNCI, Novo Nordisk Canada, Inc.; ODBF, Ontario Drug Benefit Formulary; SMBG, self‐measured blood glucose.

a Lantus (in Solostar pen) CAD 109.11 for 1500 units.

b Tresiba (in FlexTouch pen) CAD 125.28 for 1500 units.

c NovoRapid (in FlexPen) CAD 76.06 for 1500 units.

d Novofine needles, CAD 48.55 per 100 needles.

e SMBG test costs based on use of one Accu‐Chek Aviva test strip (CAD 81.73 for 100 units).

In the present analysis, it was assumed that patients using a basal insulin regimen administered one injection per day, and patients using a bolus insulin regimen administered three injections per day, with a new needle and self‐measured blood glucose test strip per injection. Insulin costs were captured as the unit cost multiplied by the mean annual dose. Complication costs were derived from the literature and were inflation‐adjusted to 2017 Canadian dollars (CAD) using the Health and Personal Care component of the Canadian Consumer Price Index (Table 2).^31^ For non‐fatal MI and non‐fatal stroke events taking place in the first annual time period, an event cost was captured in the first year, and a “state” cost was captured in the second year to reflect ongoing excess healthcare resource use after the event. Costs of severe hypoglycaemic events were captured exclusively in the year of the event and were estimated as a weighted cost of resource use, including glucagon, ambulance service, primary care, emergency care and hospital admission. Baseline utility and disutility values were based on published sources, and multiple complications were assumed to have an additive (rather than multiplicative) effect on utility (Table 2). Utilities for first‐year events of non‐fatal MI and non‐fatal stroke were half‐cycle corrected in the first year and applied fully in the second year. The disutility associated with severe hypoglycaemia was captured by multiplying an annualized disutility by the annual event rate in each time period. An overview of calculations for complication costs and QALYs in the base case analysis is presented in Table S3.

A 2‐year time horizon was selected to enable clinical model inputs to be used without extrapolation (mean [standard deviation] observation time for the DEVOTE subgroup at high risk of hypoglycaemia, 1.96 [0.41] years), obviating assumptions regarding the longevity of clinical effects. It is also highly relevant to the healthcare payer, aligning with the short‐term budget considerations commonly involved in healthcare planning.

### 2.5 Sensitivity analyses

Deterministic sensitivity analyses were conducted to identify key drivers of outcomes in the base case analysis. Annual discount rates of 0%, 3% or 5% were explored, along with variations in cardiovascular costs (20% lower, 40% lower or UK costs) or severe hypoglycaemia costs (20% lower, 40% lower or UK costs). Additional sensitivity analyses were performed, in which treatment effects (dose, hazard and rate ratios) were applied, regardless of whether they were statistically significant or not, and the glargine U100 distribution of individual MACE components was applied to both arms. Alternative disutilities were explored for cardiovascular events and severe hypoglycaemia, and analyses were conducted in which a utility associated with the flexible dosing of degludec was captured.^32^, ^33^, ^34^, ^35^

In an additional sensitivity analysis, long‐term costs and clinical outcomes with degludec vs glargine U100 were simulated over a 50‐year time horizon in the IQVIA CORE Diabetes Model version 9.0 (IQVIA, Basel, Switzerland) using cardiovascular risk equations from the United Kingdom Prospective Diabetes Study (UKPDS) Outcomes Model 2 and parameters detailed in Table S4.^36^ Cost and effectiveness outcomes, the latter expressed in QALYs, were attached to each of the four scenarios detailed in Figure S2 and did not vary between treatment arms. Distribution of patients between scenarios in the two arms was informed by the proportion of the cohort in each state at the end of the base case analysis (Table S5). The same assumptions regarding use of rescue medication were employed in both arms.

A probabilistic sensitivity analysis (PSA) was conducted to quantify the effect of statistical uncertainty around all relevant input parameters concerning cost and effectiveness outcomes (Table S6). Uncertainty was captured using normal and lognormal distributions around all key model parameters, informed by standard errors from the DEVOTE subgroup analysis. All “baseline” values (ie, those in the glargine U100 arm) were sampled from normal distributions, while all hazard, rate and dose ratios were sampled from lognormal distributions. PSA outcomes were based on 1000 model iterations with sampling from all modelled distributions in each iteration without capturing covariance. For each simulated set of values, an estimate of incremental costs and incremental QALYs was obtained.

## 3 RESULTS

### 3.1 Base case analysis

Our evaluation of discounted direct costs suggested that there would be a mean cost saving of CAD 128.58 per patient with degludec as compared to glargine U100 over a 2‐year time horizon (Table 3). The most substantial savings arose from reduced incidence of non‐fatal MI (CAD 275.67), non‐fatal stroke (CAD 266.34) and severe hypoglycaemia (CAD 199.31) with degludec vs glargine U100. These savings offset the slightly higher costs (CAD 612.76) of treatment with degludec as compared to treatment with glargine U100. Treatment with degludec was associated with a discounted improvement in quality‐adjusted life expectancy of 0.0132 QALYs relative to that with glargine U100. Quality‐of‐life benefits resulted from a reduced incidence of cardiovascular death (+0.0055 QALYs), severe hypoglycaemia (+0.0054 QALYs), non‐fatal stroke (+0.0015 QALYs) and non‐fatal MI (+0.0007 QALYs). Degludec was dominant, relative to glargine U100, resulting in improved effectiveness with lower costs (Table 3).

**Table 3:** Short‐term cost‐utility of degludec vs glargine U100 in patients with T2D at high risk of hypoglycaemia (base case analysis)

	Degludec	Glargine U100	Difference
Costs (CAD)			
Total costs	10 419.82	10 548.39	−128.58
Treatment costs			
Basal insulin	3434.10	2841.12	592.99
Bolus insulin	1631.30	1624.85	6.46
Basal needles	338.16	336.92	1.24
Bolus needles	1014.47	1010.76	3.72
Routine SMBG test	2277.00	2268.65	8.35
Costs of complications			
Non‐fatal MI	820.83	1096.50	−275.67
Non‐fatal stroke	651.26	917.61	−266.34
Severe hypoglycaemia	252.69	452.00	−199.31
Effects (QALYs)			
Total QALYs	1.4842	1.4710	0.0132
QALY break down			
Baseline	1.4969	1.4914	0.0055
Non‐fatal MI	−0.0021	−0.0029	0.0007
Non‐fatal stroke	−0.0037	−0.0053	0.0015
Severe hypoglycaemia	−0.0069	−0.0123	0.0054
ICUR (Cost/QALY)			Dominant

*Note*: Difference presented for degludec minus glargine U100. Dominant refers to improved clinical outcomes at a lower cost and is not reported per convention.

Abbreviations: CAD, Canadian dollars; glargine U100, insulin glargine 100 units/mL; ICUR, incremental cost‐utility ratio; MI, myocardial infarction; QALY, quality‐adjusted life year; SMBG, self‐measured blood glucose; T2D, type 2 diabetes.

### 3.2 Sensitivity analyses

Favourable cost‐utility results with degludec as compared to glargine U100 were insensitive to changes in most parameters (Table 4). Degludec was dominant relative to glargine U100, improving effectiveness while reducing costs, in all but four sensitivity analyses: those in which cardiovascular costs were reduced by 40%, those in which UK costs were substituted for cardiovascular complications or for severe hypoglycaemic events, and those in which additional long‐term costs and effects were included in the model. In these analyses, degludec remained highly cost‐effective as compared to glargine U100, with incremental cost‐utility ratios well below the commonly used Canadian willingness‐to‐pay threshold of CAD 50000 per QALY.^37^

**Table 4:** Results of deterministic sensitivity analyses

	ΔCosts (CAD)	ΔQALYs	ICUR (CAD per QALY)
Base case	−128.58	+0.0132	Dominant
Discount rate			
0%	−128.69	+0.0133	Dominant
3%	−128.47	+0.0131	Dominant
5%	−128.33	+0.0129	Dominant
Non‐significant treatments ratios applied	−120.69	+0.0230	Dominant
Same MACE distribution in both arms	−47.80	+0.0149	Dominant
Cardiovascular costs			
Reduced by 20%	−20.18	+0.0132	Dominant
Reduced by 40%	+88.23	+0.0132	6697.96
UK costs^a^^,^ b 38	+76.18	+0.0132	5783.45
Cardiovascular disutilities			
MI: −0.0586; stroke: −0.0462^33^	−128.58	+0.0121	Dominant
Flexible dosing utility			
0.006^34^	−128.58	+0.0246	Dominant
0.013^35^	−128.58	+0.0379	Dominant
Severe hypoglycaemia cost			
Reduced by 20%	−88.72	+0.0132	Dominant
Reduced by 40%	−48.86	+0.0132	Dominant
UK costs (CAD 737.46 per event)^a^^,^ 29	+3.26	+0.0132	247.84
Severe hypoglycaemia disutility			
−0.0118^c^^,^ 32	−128.58	+0.0088	Dominant
Long‐term extension			
Additional long‐term costs and effects^d^	+566.90	+0.0885	6407.42

*Note*: ΔCosts and ΔQALYs reported for degludec minus glargine U100. Dominant refers to improved clinical outcomes at a lower cost and is not reported per convention.

Abbreviations: Δ, difference in; CAD, Canadian dollars; ICUR, incremental cost‐utility ratio; GBP, pound sterling; glargine U100, insulin glargine 100 units/mL; MACE, major adverse cardiovascular event; MI myocardial infarction; QALY, quality‐adjusted life year; UK, United Kingdom.

a Inflation‐adjusted to 2017 prices using the hospital and community health services index from the Personal Social Services Research Unit^30^ and currency converted, based on a GBP/CAD exchange rate of 1.6720 (2017 annual average; https://www.bankofcanada.ca).

b Costs in 2017 CAD, Year 1 (Year 2): MI, 13 136.22 (3265.27); stroke, 14 127.43 (3365.46).

c Adjusted to a 1‐year time horizon, based upon one event in the past 3 months that caused a 4.7% loss of utility.

d See Table S4 for long‐term modelling extension parameters.

**Figure 1:**
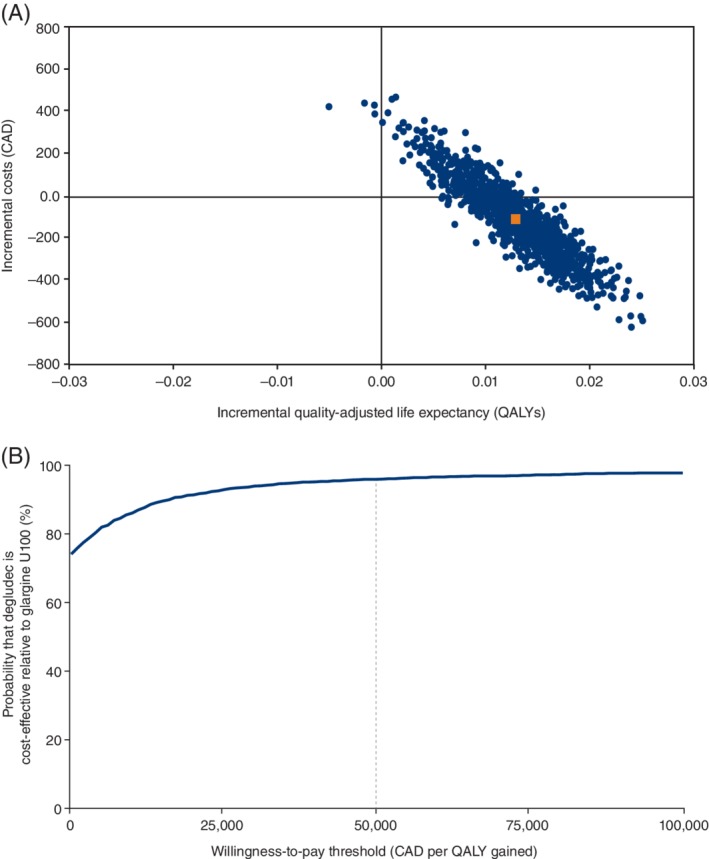
Probabilistic sensitivity analysis results: (A) cost‐utility scatterplot; (B) cost‐utility acceptability curve. (A), orange square represents the average value for incremental cost and incremental quality‐adjusted life expectancy. Abbreviations: CAD, Canadian dollars; glargine U100; insulin glargine 100 units/mL; QALY, quality‐adjusted life years

The vast majority of points on the incremental cost‐utility scatterplot were on the right‐hand side, indicating improved effectiveness with degludec as compared to glargine U100, with most points specifically in the lower right quadrant, demonstrating that costs were also lower with degludec over the 2‐year time horizon (Figure 1(A)). Using PSA results to generate a cost‐utility acceptability curve showed that there would be a 91.1% likelihood that treatment with degludec as compared to treatment with glargine U100 would be cost‐effective at a willingness‐to‐pay threshold of CAD 50000 per QALY (Figure 1(B)).

## 4 DISCUSSION

Our short‐term modelling analysis suggested that, from a Canadian public healthcare payer perspective, treatment with degludec over 2 years would be associated with improved quality‐adjusted life expectancy at lower cost, as compared to treatment with glargine U100, in patients with T2D at high risk of hypoglycaemia and cardiovascular events. These results were driven by the statistically significant reduction in risk of first MACE and severe hypoglycaemia with degludec vs glargine U100 in the DEVOTE subgroup at high risk of hypoglycaemia. Sensitivity analyses demonstrated that the results were not sensitive to changes in most input parameters, with degludec either dominant or highly cost‐effective vs glargine U100 in all analyses conducted.

Our findings share several similarities with the results of a short‐term CUA in the UK setting that was based on clinical outcomes from the DEVOTE basal‐bolus subgroup.^26^ Pollock et al. reported that degludec was dominant relative to glargine U100 over a 2‐year time horizon in patients with T2D at high cardiovascular risk who were using a basal‐bolus insulin regimen. Treatment with degludec was also associated with improved clinical outcomes (+0.0064 QALYs, discounted at 3.5% in Year 2) at cost‐neutrality (no difference in costs) as compared to treatment with glargine U100. Fractionally higher treatment costs with degludec were offset by a reduced incidence of diabetes‐related complications, particularly non‐fatal MI, and improved effectiveness was driven by lower rates of severe hypoglycaemia with degludec as compared to glargine U100.^26^ In the present analysis, cost savings with degludec were driven by lower rates of non‐fatal MI, non‐fatal stroke and severe hypoglycaemia, while increased effectiveness (+0.0132 QALYs, discounted at 1.5% in Year 2) was driven by lower risks of cardiovascular death and severe hypoglycaemia with degludec as compared to glargine U100.

Other CEAs of degludec vs glargine U100, from a public healthcare payer perspective, have reported similar findings; degludec is a dominant or cost‐effective treatment option in patients with T2D across treatment regimens, in each respective setting.^18^, ^19^, ^20^, ^21^, ^22^ To date, these have all been informed by data from phase 3, treat‐to‐target clinical trials that have focused on the short‐term effects of hypoglycaemia rates and insulin dosing over a 1‐year time horizon in a European setting.^18^, ^19^, ^20^, ^21^, ^22^ The present analysis represents the first health economic analysis of degludec vs glargine U100 for treatment of diabetes in a Canadian setting. In addition to insulin dosing and severe hypoglycaemic events, our model captured first MACE and mortality over a 2‐year period.

Severe hypoglycaemia is associated with higher incidence of various adverse outcomes in patients with diabetes, including MACE, major microvascular complications and death.^9^ It is not currently clear if there is a direct causal link between severe hypoglycaemia and adverse outcomes, or whether severe hypoglycaemia is a marker of vulnerability to a range of poor clinical outcomes.^9^ Irrespective of the nature of this relationship, our analysis demonstrates that, based on data from a subgroup analysis of DEVOTE data, treatment with degludec as compared to treatment with glargine U100 would improve quality‐adjusted life expectancy through a reduced incidence of both severe hypoglycaemia and MACE, at lower cost, in patients at high risk of hypoglycaemia in a Canadian setting.

One of the key advantages of the present analysis is its simplicity and transparency. For instance, clinical outcomes and patient characteristics were taken from a single, high‐quality data source. The base case was highly conservative, using only endpoints with significant differences between treatment arms. In addition to severe hypoglycaemia rates and insulin dosing, our short‐term CUA also captured MACE and death from other causes to provide a more in‐depth evaluation of costs and clinical outcomes without extrapolation. A further strength of this analysis was that it did not rely on long‐term cost‐effectiveness modelling and the associated assumptions concerning the progression of risk factors for diabetes‐related complications or predictions over long timeframes. Given that most complications develop over the course of decades rather than years, however, the short‐term time horizon could also be considered a limitation.

The treat‐to‐target clinical trial design renders the modelling of long‐term clinical outcomes as a function of glycaemic control inappropriate, as end‐of‐trial HbA1c tends to be similar across treatment arms. In contrast, real‐world data have demonstrated that switching to degludec from other basal insulins, including glargine U100, under conditions of routine clinical care, results in significant improvements in glycaemic control alongside other clinical benefits for T1D or T2D.^29^ By using treat‐to‐target data from a clinical trial setting, the present analysis may, therefore, underestimate the clinical benefits of degludec experienced in the real‐world clinical setting. One final limitation is that our analysis omitted microvascular complications, which can exert a large influence on healthcare costs and patient quality‐of‐life.^30^, ^38^ Microvascular events were not included as defined endpoints in the DEVOTE trial and, therefore, were omitted from our analysis in order to maintain homogeneity of clinical data. While not a limitation per se, it is worth reiterating that the present analysis was conducted in the subgroup of patients in the DEVOTE trial who were at high risk of hypoglycaemia; consequently, the results are unlikely to be applicable to other patient populations.

Our findings suggest that, in Canada, treatment with degludec over a 2‐year period is associated with improved effectiveness at lower cost as compared to treatment with glargine U100 in patients with T2D who are at high risk of hypoglycaemia and cardiovascular events. As such, our short‐term modelling analysis indicates that degludec would be an efficient use of Canadian public healthcare resources in this patient population.

## CONFLICT OF INTERESTS

R. F. P is a full‐time employee of Ossian Health Economics and Communications GmbH, which received consultancy fees from Novo Nordisk to construct the model and to conduct the analyses. S. H. reports consultancy fees from Eli Lilly and Co., Novo Nordisk and Takeda; has participated in speakers' bureaus for Novo Nordisk, Eli Lilly and Co, Merck Sharp and Dohme, Takeda and AstraZeneca; and has served on advisory panels for Novo Nordisk, Eli Lilly and Co., Boeringher Ingelheim, Sanofi Aventis, Zealand Pharma and UN‐EEG. T. R. P. is an employee of CBmed; he reports consultancy fees from Arecor, AstraZeneca, Eli Lilly and Co., Novo Nordisk and Sanofi and has received research support from Novo Nordisk and AstraZeneca. V. W. has served on advisory boards and speakers' bureaus for Novo Nordisk, Eli Lilly and Co., Merck Sharp and Dohme, Boehringer Ingelheim, BMS, Sanofi, AstraZeneca, Johnson and Johnson, Roche and Abbott. J. G., N. H., M. L. and D.T. are employees of Novo Nordisk; and D. T. and J. G. hold shares/stocks in Novo Nordisk. B. Z. has received consultancy fees from Novo Nordisk, Boehringer Ingelheim, AstraZeneca, Eli Lilly and Co., Janssen, Sanofi, Merck and Abbott.

## AUTHOR CONTRIBUTIONS

R. F. P. is the guarantor of this work and, as such, had full access to all data in the study and takes responsibility for the integrity of the data and the accuracy of data analysis. All authors confirm that they meet the International Committee of Medical Journal Editors (ICJME) uniform requirements for authorship and that they have contributed to critical analysis and interpretation of the data, to drafting and/or critically revising the article, and they share final responsibility for the content of the manuscript, as well as the decision to submit for publication.

The datasets generated and/or analysed during the current study are available from the corresponding author upon reasonable request.

## Supporting information


**FIGURE S1** Schematic illustration of the cost‐utility model
**Figure S2** Long‐term modelling extension scenarios
**Table S1** Cox regression model of time to first event of severe hypoglycaemia in DEVOTE (N = 7637)
**Table S2** Baseline characteristics for the DEVOTE subgroup at high risk of hypoglycaemia
**Table S3** Overview of cost and QALY calculations for complications
**Table S4** Long‐term extension modelling parameters (at baseline)
**Table S5** Overview of the long‐term extension sensitivity analysis methodology and simulated results
**Table S6** Parameter inputs for the probabilistic sensitivity analysis
