# Supplementary material for: Short‐term cost‐utility of degludec versus glargine U100 for patients with type 2 diabetes at high risk of hypoglycaemia and cardiovascular events: A Canadian setting (DEVOTE 9)
Source: Diabetes Obes Metab. 2019 Apr 14;21(7):1706–14. doi: 10.1111/dom.13730 (PMC6618053; doi:10.1111/dom.13730)
Supplement: Supplementary file 1 — FIGURE S1 Schematic illustration of the cost‐utility model Figure S2 Long‐term modelling extension scenarios Table S1 Cox regression model of time to first event of severe hypoglycaemia in DEVOTE (N = 7637) Table S2 Baseline characteristics for the DEVOTE subgroup at high risk of hypoglycaemia Table S3 Overview of cost and QALY calculations for complications Table S4 Long‐term extension modelling parameters (at baseline) Table S5 Overview of the long‐term extension sensitivity analysis methodology and simulated results Table S6 Parameter inputs for the probabilistic sensitivity analysis [file DOM-21-1706-s001.docx]

Supporting information for:

Short-term cost-utility of degludec versus glargine U100 for patients with type 2 diabetes at high risk of hypoglycaemia and cardiovascular events – a Canadian setting (DEVOTE 9)

Richard F Pollock MSci^1^, Simon Heller MD^2^, Thomas R Pieber MD^3^, Vincent Woo MD^4^, Jens Gundgaard PhD^5^, Nino Hallén MSc^5^, Maria Luckevich MSc^6^, Deniz Tutkunkardas MD^5^, Bernard Zinman MD^7^, on behalf of the DEVOTE study group

^1^Ossian Health Economics and Communications GmbH, Basel, Switzerland; ^2^University of Sheffield, Sheffield, UK; ^3^Medical University of Graz, Graz, Austria; ^4^University of Manitoba, Winnipeg, Canada; ^5^Novo Nordisk A/S, Søborg, Denmark; ^6^Novo Nordisk Canada Inc., Mississauga, Canada; ^7^Lunenfeld-Tanenbaum Research Institute, Mount Sinai Hospital, University of Toronto, Toronto, Canada

# 1. SUPPORTING METHODS

DEVOTE enrolled patients with type 2 diabetes (T2D) treated with at least one oral or injectable antihyperglycaemic agent and HbA1c ≥7%. Patients with HbA1c <7% were eligible if they were receiving ≥20 units/day of basal insulin. Two groups of patients were eligible for the trial: patients aged ≥50 years old with at least one co-existing cardiovascular or renal condition and patients aged ≥60 years old with at least one cardiovascular risk factor. Please refer to the primary manuscript for further details.^1^

## 1.1 Subgroup analyses for the DEVOTE subgroup at high hypoglycaemia risk

A series of subgroup analyses were conducted for degludec versus glargine U100. Time to first MACE was analyzed using a Cox proportional-hazards regression model on the composite MACE endpoint. Death from other causes was analyzed using a Cox proportional-hazards regression model. The number of severe hypoglycaemic events was analyzed using a negative binomial regression model with a log-link function and log (duration of observation time) as offset. Insulin dose (units/kg) was log transformed and analyzed using a mixed model for repeated measures (MMRM) within patients using an unstructured residual covariance matrix among visits. Visit interactions with age, dose at baseline, body mass index, alternative titration target (Yes/No) and treatment were included in the model as fixed effect covariates. Change in HbA1c from baseline to the 24-month visit was analyzed using an MMRM within patients; an unstructured residual covariance matrix among visits at 6, 12 and 24 months of the trial; interactions between visit and treatment, and visit and baseline HbA1c were included as fixed effects in the model.

### **FIGURE S1** Schematic illustration of the cost-utility model


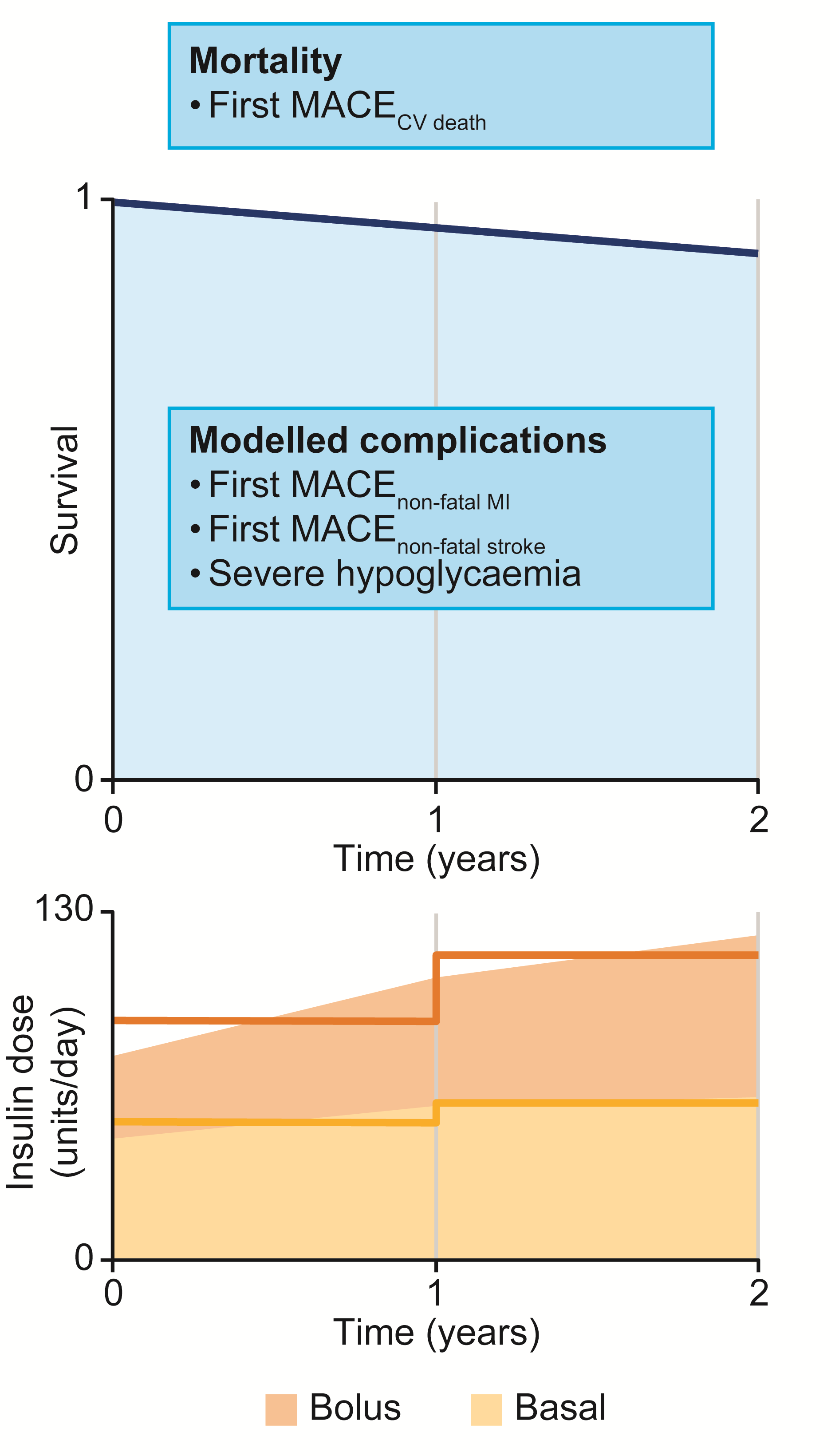


Diagram shows the model as run for each treatment arm (degludec and glargine U100). The model used relative rates (hazard, rate and dose ratios) from regression analyses to derive the rates and doses in the degludec arm (observed glargine U100 rates and doses are displayed in the figure). The basal insulin dose was modelled as the arithmetic mean of the start- and end-of-year glargine U100 basal doses to approximate the area under the curve, adjusted for survival in each annual time period. This was repeated for bolus insulin, but the mean annual bolus dose was estimated by multiplying the proportion of patients receiving bolus insulin at baseline, 12 or 24 months by the mean bolus dose for each time point. Severe hypoglycaemia was defined according to the American Diabetes Association definition as an event requiring third-party assistance.^2^

Abbreviations: CV, cardiovascular; glargine U100; insulin glargine 100 units/mL; MACE, major adverse cardiovascular event; MI, myocardial infarction.

### **FIGURE S2** Long-term modelling extension scenarios

**
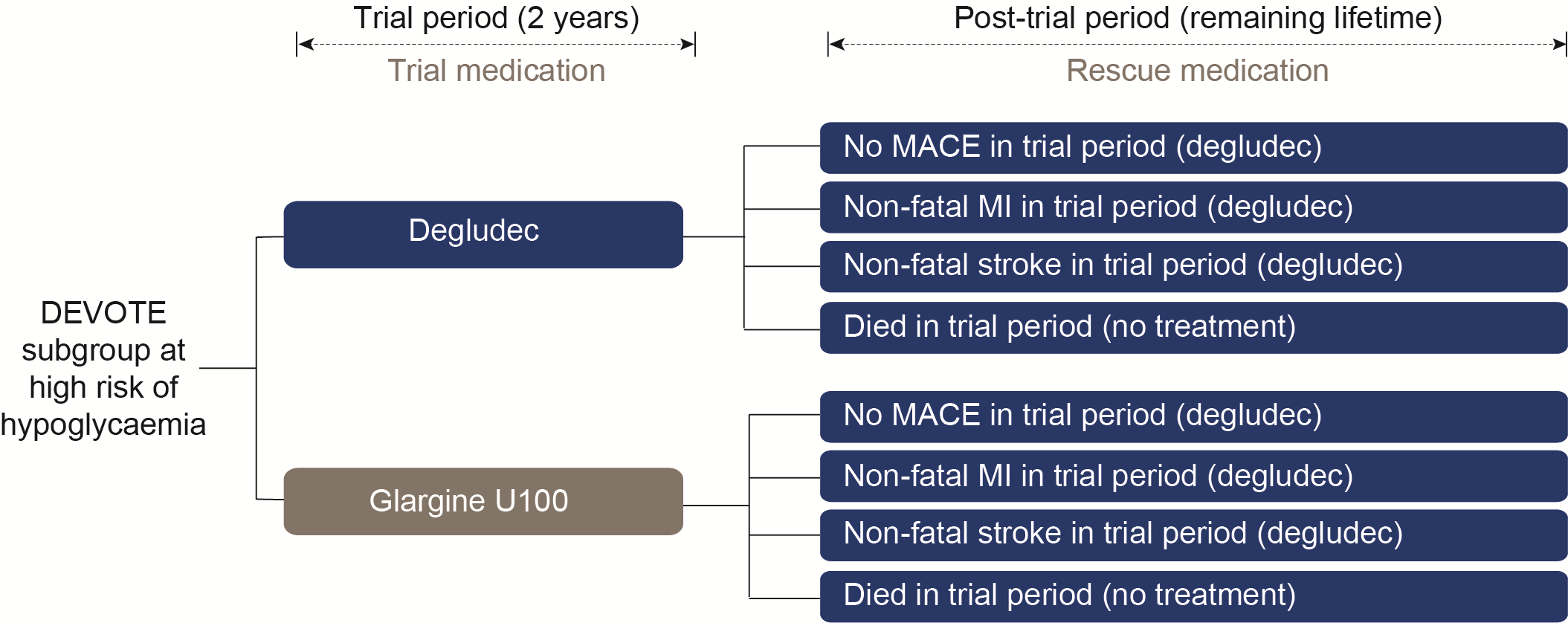
**

Scenarios were simulated in the IQVIA CORE Diabetes Model version 9.0 (IQVIA, Basel, Switzerland), based on the baseline characteristics in Table S4 with no changes in HbA1c or treatment costs as in the degludec arm, based on end-of-trial doses. The four scenarios included no first MACE experienced in the trial period, non-fatal MI (from first MACE) in trial period, non-fatal stroke (from first MACE) in trial period, died in trial period (from cardiovascular death or other causes). The four scenarios are the same for the two arms in terms of costs and QALYs, with only the distribution of scenarios between the two arms varied (further details provided in Table S5).

Abbreviations: Glargine U100, insulin glargine 100 units/mL; MACE, major adverse cardiovascular event; MI, myocardial infarction; QALY, quality-adjusted life year.

**Table S1** Cox regression model of time to first event of severe hypoglycaemia in DEVOTE (N = 7637)

| **Variable** | **Parameter estimate** | **SE** | ***P*-value** | **Hazard ratio** |
| --- | --- | --- | --- | --- |
| Previous simplified insulin regimen (Insulin naïve) | -0.076 | 0.174 | 0.665 | 0.927 |
| Previous simplified insulin regimen (Basal–bolus) | 0.552 | 0.111 | < 0.0001 | 1.737 |
| Sex (Male) | -0.345 | 0.097 | 0.0004 | 0.708 |
| Baseline HbA1c | 0.050 | 0.030 | 0.092 | 1.051 |
| Baseline diabetes duration | 0.020 | 0.005 | 0.0002 | 1.020 |
| Baseline age | 0.017 | 0.007 | 0.015 | 1.017 |

| **Model fit statistics**  -2 Log L: 7339.074^†^, 7255.888^‡^  AIC: 7339.074^†^, 7267.888^‡^  SBC: 7339.074^†^, 7292.257^‡^ |
| --- |
| **Testing the global null hypothesis (BETA = 0)**  Likelihood ratio: 83.186*  Score: 84.763*  Wald: 83.029* |

A total of 7548 patients had available data and were included in the model. High risk of severe hypoglycaemia was based on scores ≥75^th^ percentile of model scores (2.17).

**P* < 0.0001.

^†^Without covariates.

^‡^With covariates.

Abbreviations: AIC, Akaike’s information criterion; L, likelihood; SBC, Schwarz bayesian (information) criterion; SE, standard error.

**TABLE S2** Baseline characteristics for the DEVOTE subgroup at high risk of hypoglycaemia

|  | **Degludec** | **Glargine U100** | **Total** |
| --- | --- | --- | --- |
| n | 956 | 931 | 1887 |
| Age, years | 67.9 (7.2) | 68.4 (7.3) | 68.1 (7.3) |
| **Gender, n (%)** |  |  |  |
| Female | 610 (63.8) | 605 (65.0) | 1215 (64.4) |
| Male | 346 (36.2) | 326 (35.0) | 672 (35.6) |
| Body weight, kg | 95.2 (21.3) | 94.1 (22.0) | 94.6 (21.6) |
| BMI, kg/m^2^ | 34.7 (7.0) | 34.4 (7.1) | 34.6 (7.0) |
| Duration of diabetes, years | 23.6 (9.1) | 23.4 (9.6) | 23.5 (9.3) |
| HbA1c, % | 8.66 (1.66) | 8.63 (1.79) | 8.64 (1.73) |
| HbA1c, mmol/mol | 71.2 (18.1) | 70.8 (19.6) | 71.0 (18.9) |
| **Insulin treatment regimen, n (%)** |  |  |  |
| Naïve | 5 (0.5) | 7 (0.8) | 12 (0.6) |
| Basal only | 50 (5.2) | 50 (5.4) | 100 (5.3) |
| Basal–bolus (including premix) | 847 (88.6) | 819 (88.0) | 1666 (88.3) |
| Bolus only | 54 (5.6) | 55 (5.9) | 109 (5.8) |
| **Region, n (%)** |  |  |  |
| Europe | 98 (10.3) | 78 (8.4) | 176 (9.3) |
| North America | 730 (76.4) | 724 (77.8) | 1454 (77.1) |
| South America | 39 (4.1) | 40 (4.3) | 79 (4.2) |
| Asia (excluding India) | 34 (3.6) | 31 (3.3) | 65 (3.4) |
| India | 25 (2.6) | 19 (2.0) | 44 (2.3) |
| Africa | 30 (3.1) | 39 (4.2) | 69 (3.7) |
| **Race, n (%)** |  |  |  |
| White | 725 (75.8) | 705 (75.7) | 1430 (75.8) |
| Black or African American | 124 (13.0) | 133 (14.3) | 257 (13.6) |
| Asian | 75 (7.8) | 61 (6.6) | 136 (7.2) |
| American Indian or Alaska Native | 4 (0.4) | 2 (0.2) | 6 (0.3) |
| Native Hawaiian or Other Pacific Icelander | 2 (0.2) | 4 (0.4) | 6 (0.3) |
| Other | 26 (2.7) | 26 (2.8) | 52 (2.8) |
| **Ethnicity, n (%)** |  |  |  |
| Hispanic or Latino | 116 (12.1) | 121 (13.0) | 273 (12.6) |
| Non-Hispanic or Latino | 840 (87.9) | 810 (87.0) | 1650 (87.4) |
| **CV risk group, n (%)** |  |  |  |
| Established CVD/CKD | 837 (87.6) | 807 (86.7) | 1644 (87.1) |
| Risk factors for CVD | 116 (12.1) | 123 (13.2) | 239 (12.7) |
| **Exposure** |  |  |  |
| PYO | 1.98 (0.39) | 1.95 (0.42) | 1.96 (0.41) |
| PYE | 1.75 (0.52) | 1.71 (0.55) | 1.73 (0.54) |

Data are mean (standard deviation) unless otherwise stated.

Abbreviations: %, percentage of patients; BMI, body mass index; CKD, chronic kidney disease; CVD, cardiovascular disease; glargine U100, insulin glargine 100 units/mL; n, number of patients; PYE, patient-year of exposure; PYO, patient-year of observation.

**TABLE S3** Overview of cost and QALY calculations for complications

|  | **Time period 1** | **Time period 2^†^** |
| --- | --- | --- |
| **Cost of complications (CAD)** |  |  |
| Non-fatal MI, year 1 | 19,806.63 | 19,806.63 |
| Non-fatal MI, year 2 | N/A | 3097.33 |
| Non-fatal stroke, year 1 | 26,978.80 | 26,978.80 |
| Non-fatal stroke, year 2 | N/A | 3743.24 |
| Severe hypoglycaemia (per event) | 2178.62 | 2178.62 |
| Cardiovascular death | Assumed to be 0 | Assumed to be 0 |
| **Effects (QALYs)^‡^** |  |  |
| Alive | 0.785 × proportion of time alive | 0.785 × proportion of time alive |
| Non-fatal MI, year 1 | –0.055 × 0.5^§^ | –0.055 × 0.5^§^ |
| Non-fatal MI, year 2 | N/A | –0.055 × proportion of time alive |
| Non-fatal stroke, year 1 | –0.164 × 0.5^§^ | –0.164 × 0.5^§^ |
| Non-fatal stroke, year 2 | N/A | –0.164 × proportion of time alive |
| Severe hypoglycaemia | –0.0592 × annual event rate | –0.0592 × annual event rate |
| Cardiovascular death^¶^ | Assumed to be 0 | Assumed to be 0 |

Based on the input parameters presented in Table 2. Year 1 refers to the ‘event year’ i.e. the time period that the complication takes place in, while year 2 refers to the ‘subsequent year’ i.e. the subsequent time period following a complication in the preceding time period.

^†^All values in time period 2 discounted by 1.5% before summing all costs and QALYs.

^‡^Values presented are utilities (QALYs are determined once calculations [described in the table] are completed and utilities summed).

^§^Multiplied by 0.5 to half-cycle correct.

^¶^Effects captured in ‘Alive’ QALYs.

Abbreviations: CAD, Canadian dollar; MI, myocardial infarction; N/A, not applicable; QALY, quality-adjusted life year.

### **TABLE S4** Long-term extension modelling parameters (at baseline)

| **Variable** | **Parameter** |  | **Units** |
| --- | --- | --- | --- |
| **Patient demographics** |  |  |  |
| Age^1^ | 70.1 |  | years |
| Duration of diabetes^†^ | 25.5 |  | years |
| Gender (male) | 35.6 |  | % |
| **Risk factors** |  |  |  |
| HbA1c^‡^ | 7.68 |  | % |
| Systolic blood pressure | 136.2 |  | mmHg |
| Diastolic blood pressure | 73.7 |  | mmHg |
| Total cholesterol | 168.22 |  | mg/dL |
| HDL-cholesterol | 47.73 |  | mg/dL |
| LDL-cholesterol | 86.53 |  | mg/dL |
| Triglycerides | 178.91 |  | mg/dL |
| Body mass index | 34.6 |  | kg/m^2^ |
| eGFR | 61.08 |  | ml/min/1.73m^2^ |
| Haemoglobin count | 12.94 |  | gr/dL |
| White blood cell count^§^ | 6.8 |  | 10^6^/mL |
| Heart rate^¶^ | 72.3 |  | bpm |
| Smokers | 7.4 |  | % |
| **Racial characteristics**^††^ |  |  |  |
| White | 77.93 |  | % |
| Black | 14.01 |  | % |
| Native American | 0.33 |  | % |
| Asian/Pacific Islander | 7.74 |  | % |
| **CVD complication history** |  |  |  |
| Myocardial infarction^‡‡^ | 31.5 |  | % |
| Angina | 11.9 |  | % |
| Peripheral vascular disease^§§^ | 32.5 |  | % |
| Stroke^¶¶^ | 18.6 |  | % |
| Heart failure | 18.7 |  | % |
| Atrial fibrillation | 10.3 |  | % |
| Left ventricular hypertrophy | 11.1 |  | % |
| **Renal complications** |  |  |  |
| Microalbuminuria^†††^ | 24.4 |  | % |
| Gross proteinuria | 0 |  | % |
| End-stage renal disease | 0 |  | % |
| **Retinopathy complications** |  |  |  |
| Background diabetic retinopathy^‡‡‡^ | 21.7 |  | % |
| **Foot ulcer complications** |  |  |  |
| Uninfected ulcer^§§§^ | 10.3 |  | % |
| History of amputation | 1.7 |  | % |
| Macular oedema | 0.7 |  | % |
| Cataract | 6.5 |  | % |
| Neuropathy | 17.9 |  | % |
| Depression^¶¶¶^ | 0 |  | % |

All parameters are from the DEVOTE subgroup at high risk of hypoglycaemia at baseline, if nothing else is noted.

^†^Baseline value plus 2 years.

^‡^Based on 8.64% minus 0.96% (degludec HbA1c change from baseline).

^§^CORE default value.

^¶^Pulse.

^††^Data adapted to fit CORE requirement that racial characteristics sum to 100%: Asians and native Hawaiians (or other Pacific Islander) were aggregated and the relative distribution calculated based on White, Black (or African American), Native American, Asian/Pacific Islander.

^‡‡^Set to 100% for scenarios of patients experiencing first non-fatal myocardial infarction in the trial.

^§§^Peripheral artery disease.

^¶¶^Set to 100% for scenarios of patients experiencing first non-fatal stroke in the trial.

^†††^Aggregated value for microalbuminuria and gross proteinuria (disaggregated numbers could not be extracted).

^‡‡‡^Aggregated value for background, proliferative diabetic retinopathy and severe vision loss (disaggregated numbers could not be extracted).

^§§§^Aggregated value for uninfected, infected and healed ulcer (disaggregated numbers could not be extracted).

^¶¶¶^Assumed 0.

Abbreviations: CVD, cardiovascular disease; eGFR, estimated glomerular filtration rate; HDL, high-density lipoprotein; LDL, low-density lipoprotein.

**TABLE S5** Overview of the long-term extension sensitivity analysis methodology and simulated results

| **Simulation arm** | **Long-term extension scenario** | **Baseline characteristics** | **Treatment costs**  **and severe hypoglycaemia rate** | | **Distribution of scenarios after 2 years (%)** | **Simulated results, years 3+**^†^ | |
| --- | --- | --- | --- | --- | --- | --- | --- |
|  |  |  |  |  |  | **Costs (CAD)** | **QALE (QALYs)** |
| Degludec | No MACE in trial period | Table S4 | As degludec | 86.1 | | 124,373.7 | 6.873 |
|  | Non-fatal MI in trial period | Table S4^‡^ | As degludec | 3.8 | | 133,550.3 | 6.091 |
|  | Non-fatal stroke in trial period | Table S4^§^ | As degludec | 2.2 | | 127,246.3 | 4.683 |
|  | Died during trial period | No simulation | N/A | 7.8 | | 0.0 | 0.0 |
|  |  |  |  | Weighted average^¶^ | | 115,039.8 | 6.255 |
| Glargine U100 | No MACE in trial period | Table S4 | As degludec | 83.2 | | 124,373.7 | 6.873 |
|  | Non-fatal MI in trial period | Table S4^‡^ | As degludec | 5.1 | | 133,550.3 | 6.091 |
|  | Non-fatal stroke in trial period | Table S4^§^ | As degludec | 3.2 | | 127,246.3 | 4.683 |
|  | Died during trial period | No simulation | N/A | 8.5 | | 0.0 | 0.0 |
|  |  |  |  | Weighted average^¶^ | | 114,323.3 | 6.178 |

Scenarios (detailing in Figure S2) were simulated using the IQVIA CORE Diabetes Model version 9.0 (IQVIA, Basel, Switzerland).

^†^Further discounted by 1.5% to year 1.

^‡^With MI history set to 100%.

^§^With stroke history set to 100%.

^¶^Weighted average of simulated costs and QALYs in years 3+ (weighted by the proportion of the cohort in each state after simulating 2 time periods [years]).

Abbreviations: CAD, Canadian dollars; Glargine U100, insulin glargine 100 units/mL; MACE, major adverse cardiovascular event; MI, myocardial infarction; N/A, not applicable; QALE, quality-adjusted life expectancy; QALY, quality-adjusted life year.

### **TABLE S6** Parameter inputs for the probabilistic sensitivity analysis

|  | **Parameter** | | **SE** | |  | **Distribution** | | |  |  |
| --- | --- | --- | --- | --- | --- | --- | --- | --- | --- | --- |
| **Treatment ratio (degludec/glargine U100)** | |  | |  | | |  |  | | |
| *Complications* |  | |  | |  |  | | | |  |
| First MACE | 0.757 | | 0.13 | |  | Lognormal | | | |  |
| Severe hypoglycaemia | 0.557 | | 0.182 | |  | Lognormal | | | |  |
| All-cause mortality | 0.721 | | 0.235 | |  | Lognormal | | | |  |
| *Basal dose* |  | |  | |  |  | | | |  |
| Basal dose, 12 months | 1.049 | | 0.022 | |  | Lognormal | | | |  |
| Basal dose, 24 months | 1.087 | | 0.030 | |  | Lognormal | | | |  |
| *Bolus dose* |  | |  | |  |  | | | |  |
| Bolus dose, 12 months | 0.959 | | 0.033 | |  | Lognormal | | | |  |
| Bolus dose, 24 months | 0.946 | | 0.047 | |  | Lognormal | | | |  |
| **Disutility** |  | |  | |  |  | | | |  |
| Severe hypoglycaemia^3^ | 0.0592 | | 0.0050 | |  | Normal | | | |  |
| Non-fatal myocardial infarction^4^ | 0.055 | | 0.0064 | |  | Normal | | | |  |
| Non-fatal stroke^4^ | 0.164 | | 0.0298 | |  | Normal | | | |  |

Disutility SEs were calculated from reported 95% CI using the following formula: SE = lower 95% CI minus upper 95% CI/2 multiplied by 1.96, assuming that sampling was normally distributed.

Abbreviations: CI, confidence interval; glargine U100, insulin glargine 100 units/mL; MACE, major adverse cardiovascular event; SE, standard error.

### **References**

1. Marso SP, McGuire DK, Zinman B, et al. Efficacy and Safety of Degludec versus Glargine in Type 2 Diabetes. *N Engl J Med.* 2017;377(8):723–732.

2. Seaquist ER, Anderson J, Childs B, et al. Hypoglycemia and diabetes: a report of a workgroup of the american diabetes association and the endocrine society. *Diabetes Care.* 2013;36(5):1384–1395.

3. Harris S, Mamdani M, Galbo-Jorgensen CB, Bogelund M, Gundgaard J, Groleau D. The effect of hypoglycemia on health-related quality of life: Canadian results from a multinational time trade-off survey. *Can J Diabetes.* 2014;38(1):45–52.

4. Clarke P, Gray A, Holman R. Estimating utility values for health states of type 2 diabetic patients using the EQ-5D (UKPDS 62). *Medical decision making : an international journal of the Society for Medical Decision Making.* 2002;22(4):340–349.
